# Conceptualizing access to community-based supports from the perspectives of people living with young onset dementia, family members and providers

**DOI:** 10.1177/14713012221138145

**Published:** 2022-11-14

**Authors:** Sheila Novek, Verena Menec

**Affiliations:** School of Nursing, University of British Columbia, Vancouver, Canada; Department of Community Health Sciences, Rady Faculty of Health Sciences, 423134 University of Manitoba, Winnipeg, Canada

**Keywords:** dementia, young onset dementia, support services, access, candidacy, semi-structured interviews, thematic analysis, Canada

## Abstract

People living with young onset dementia and their families have significant support needs, but experience difficulties accessing services. This study explored the process of accessing community-based services drawing on semi-structured interviews with people living with dementia, family members and providers in Winnipeg, Canada. Data analysis involved a combination of inductive coding and theoretical analysis using the candidacy framework as a conceptual lens. Forced to navigate services that do not recognize people with young onset dementia as a user group, participants experienced ongoing barriers that generated continuous work and stress for families. Access was constrained by information resources geared towards older adults and restrictive eligibility criteria that constructed people with young onset dementia as “not impaired enough” or “too impaired”. At the organizational level, fragmentation and underrepresentation of young onset dementia diminished access. Our findings underscore the need for continuous, coordinated supports alongside broader representation of young onset dementia within research, policy, and practice. We conclude with a discussion of how the candidacy theory could be extended to account for the social and political status of user groups.

## Introduction

A growing body of evidence suggests that individuals and families affected by young onset dementia have significant support needs, but experience difficulties accessing services (Bakker et al., 2013, 2014; Greenwood & Smith, 2016; Millenaar et al., 2016; Svanberg et al., 2011). Despite growing calls for specialized supports, people with young onset dementia remain underrepresented in dementia policies and programming (Carter et al., 2018). As a result, those living with young onset dementia must navigate systems and services designed for older adults, and emerging research points to significant access barriers (Greenwood & Smith, 2016; Millenaar et al., 2016) and low levels of service use (Bakker et al., 2013; Cations et al., 2017; Gibson et al., 2014; O'Connor et al., 2021). Although research on young onset dementia is burgeoning, it remains at an “early stage theoretically and methodologically” (Spreadbury & Kipps, 2019: 137). Given the complexity of access issues, and the paucity of theoretically informed research on the subject, the aim of this study was to explore the process of accessing community-based supports from the perspectives of people living with young onset dementia, family members and providers, drawing on the theoretical construct of candidacy (Dixon-Woods et al., 2005, 2006).

## Young Onset Dementia and Access To Community-Based Services

The term young onset dementia, or early-onset dementia, refers to dementia that develops within younger age groups, typically defined as before the age of 65 (Carter et al., 2018; Draper & Withall, 2016). Young onset dementia is not a diagnostic category, and age is not used to determine dementia subtypes (Ballenger, 2006). Instead, the term is increasingly used by those affected by the condition, advocacy groups, policy makers and researchers to draw attention to an overlooked population with particular characteristics, needs and barriers to services (Carter et al., 2018; Koopmans & Rosness, 2014). For example, the types of dementia diagnosed in younger age groups are more diverse compared to older adults, so illness progression and support needs vary widely (Mayerhofer et al., 2020). Since young onset dementia strikes at midlife, people with young onset dementia are often forced to give up employment. They may also have dependent children living at home, raising concerns about financial ramifications and the impacts on children and young adults (Millenaar et al., 2014). Stamou et al. (2020) argue that community supports, such as home supports, recreational programs and psychosocial supports, provide a critical safety net for people affected by young onset dementia. Previous studies have shown that effective support services help people affected by young onset dementia understand and accept their diagnosis (Millenaar et al., 2018; Stamou et al., 2020); secure financial benefits and legal advice (Mayrhofer et al., 2018; Wheeler et al., 2015); overcome stigma and establish new social connections (Beattie et al., 2004; Mayrhofer et al., 2018; Stamou et al., 2020); participate in meaningful activities (Beattie et al., 2004; Richardson et al., 2016; Roach & Drummond, 2014; Van Vliet et al., 2017); engage in activism and social citizenship (Phinney et al., 2016; Stamou et al., 2020); and remain living at home for longer (Mayrhofer et al., 2018).

Despite high needs for support services, there is a growing consensus that people with young onset dementia experience access barriers due to age-restrictions (Cations et al., 2017); the absence of specialized supports (Bayly et al., 2021; Cartwright et al., 2021; Mayrhofer et al., 2018); the lack of tailored information (Cations et al., 2017); and service fragmentation (Mayrhofer et al., 2018). Research on this topic, however, remains conceptually underdeveloped. Following a review of models of service access, Pratt et al. (2006) call for more dynamic conceptualizations of dementia care access that integrate individual, social-interactional and wider contextual dimensions of service access and utilization.

## Conceptualizing Service Access: The Candidacy Framework

In the present analysis, we applied the lens of the candidacy framework (Dixon-Woods et al., 2005, 2006) to examine these multifaceted dimensions of service access. The concept of candidacy, “describes the ways in which eligibility for medical attention and intervention is jointly negotiated between individuals and health services” (Dixon-Woods et al., 2006: 7). Access to care is conceptualized as a dynamic and contingent process of negotiation, constructed through interactions between individuals seeking care (patients, family members or carers), providers and institutions. These social interactions are shaped by organizational conditions, as well as the wider social and policy contexts. Although the candidacy framework was originally intended to theorize health care access, it has since been applied to the study of social service use among diverse populations (Green et al., 2020; Koehn, 2009; Mackenzie et al., 2013).

The framework identifies five dimensions of candidacy that represent transition points in the process of candidacy negotiation (Dixon-Woods et al., 2005). The first dimension–*identification*–refers to the process through which individuals (service users, family members or carers) recognize a need that would benefit from a service. Once an individual has identified a need for services, they must gain entry to that service. The next dimension–*navigation*–refers to the work required to access and maintain service use. After finding an entry point, individuals or their family member must interact with providers to assert a claim for candidacy. The third and fourth dimensions–*appearances and adjudications*–refer to the ways that individuals present their candidacy, and the judgements made about that candidacy by professionals (Dixon-woods, et al., 2006). After qualifying for a service, an individual may decide to accept or decline the offer. Thus, the fifth dimension–*offers and resistance*–refers to the services offered following adjudications of candidacy, as well as the decisions of services users to engage or refuse such services. Each dimension is shaped by local *operating conditions*–that is the organizational and wider contextual factors that influence candidacy negotiations.

## Methods

This study involved a combination of sequential, semi-structured interviews with people living with young onset dementia and family members to explore their perspectives and experiences (Kelly, 2011); and one-time, semi-structured interviews with health and service providers. Multiple stakeholders were included to situate individual and family experiences within the broader context of health and social care, while also ensuring that the voices of people living with young onset dementia were included.

### Study Context

The study was conducted in Winnipeg, Canada. Winnipeg is a midsized city and capital of the province of Manitoba. In Canada, medically necessary hospital and physician services are universally insured in all provinces, but community support services such as home care, and social programming are not (Canadian Academy of Health Sciences, 2019). In Manitoba, home support services are publicly administered and available free of charge, based on assessed need and service availability. Adult day services, which provide social and recreational opportunities, are publicly administered and delivered for a fee. Other community-based dementia supports such as educational sessions and peer support groups are delivered by non-profit organizations. Like most cities in Canada, Winnipeg does not have specialized services for people with young onset dementia, apart from a support group for carers run by the Alzheimer Society, a non-profit organization.

### Participant Recruitment

Following approval from the Health Research Ethics Board of the University of Manitoba, we recruited a purposive sample of people living with dementia, family members and providers, between February and November 2017. *People with dementia and family members* were recruited through multiple avenues (support groups, clinicians, advertisements, and a study website). The first author met with three support groups hosted by the Alzheimer Society to discuss the study and distribute recruitment materials (i.e., brochures with information about the study, contact details, and the study website). Participants were also recruited through specialist physicians and clinicians who work with patients with young onset dementia. Interested clinicians were provided with study brochures to distribute to patients or family members. Advertisements for the study were also distributed through local community organizations such as the Alzheimer Society. The study website contained information about the study, eligibility criteria, study procedures, and contact information. Family members of individuals who had young onset dementia, or had passed away from young onset dementia within the past year, were eligible for this study. Multiple members of the same family were invited to participate.

*Providers* who work with this population were identified through websites for health and social care organizations, and subsequent snowball sampling. Providers who work with people living with dementia were contacted directly by phone, email or mail and invited to participate in the study. Employing a snowball sampling technique, participants were asked to provide contact information for other potential participants. Recruitment off all participant groups ceased in October 2017 after both authors determined a sufficient level of saturation had been achieved. For further details on recruitment procedures, see (Novek & Menec, 2021).

### Participants

In total, six people with young onset dementia and 14 family members of people affected by young onset dementia participated in the study (11 spouses, two adult children and one niece). Six family members participated on their own, with no other family members participating in the study. The other 14 participants comprised four couples (person with dementia and their spouse) and two families of three (person with dementia, spouse, and adult child).

Among participants with dementia, four were diagnosed with Alzheimer’s disease, one had Lewy body dementia, and one had an unknown subtype; there were four males and two females ranging in age from 57 to 66 years old. Among family members, half were male, half were female and their ages ranged from 20 to 76 years. Their relatives had diagnoses of Alzheimer’s disease (n = 10), Lewy body dementia (n = 1), frontotemporal dementia (n = 1) and unknown dementia (n = 2). In two cases, their relative with dementia had died within the past year.

Providers included 16 individuals from a variety of professions: 1) eight physicians with expertise in young onset dementia; 2) four health care providers involved in diagnosing or supporting people with young onset dementia and/or family members (e.g., social workers); and 3) four service providers who work with people with dementia (e.g., support group facilitators).

### Interview Procedures

All participants provided informed consent before participating in interviews, and steps were taken to ensure a thorough and inclusive consent process. Participants with dementia were asked if they wanted to conduct the interviews by themselves, or with a support person present, and all six chose to have their spouses accompany them. The first author read the consent form out loud and asked checking questions to ensure understanding. Participants with dementia had the option to provide verbal or written consent, and all six chose to provide verbal consent. Ongoing process consent was also practiced throughout interviews by seeking ongoing verbal consent, restating the purpose of the research, answering questions, and responding to signs of discomfort (Dewing, 2007; Novek & Wilkinson, 2019). Family members and providers all provided written informed consent.

Semi-structured interviews with people living with young onset dementia and family members of individuals affected by young onset dementia were conducted at two points in time. Sequential interviews were conducted to build rapport, to add depth and detailed description, and to enable exploration of emergent themes (Charmaz, 2001). The first interview was used to explore participants’ experiences of the diagnostic process and community-based supports, as well as the impact of life with dementia on the family. Family members were also asked to complete a brief demographic questionnaire. Follow-up interviews were conducted between three to 6 months after the initial interview and explored subsequent experiences with health and support services, as well as follow-up questions based on the topics that arose during the previous interview. Interviews took place in participants’ homes (n = 16), at an alternate location (n = 3), or over the phone (n = 1) and ranged from 23 to 133 min.

Providers were asked about their experiences providing care to people with young onset dementia and/or family members, strengths and gaps within current services, and the factors that affect access to dementia supports. Depending on the preference of participants, interviews took place in participants’ offices (n = 13) or over the phone (n = 3), and ranged from 22 and 103 min. All interviews were audio-recorded, transcribed by a professional transcriptionist, and anonymized.

### Data Analysis

Data analysis involved an iterative process of inductive, thematic analysis (Braun & Clarke, 2006), and deductive, theoretical analysis using the candidacy framework as a conceptual lens. Both authors read all transcripts to get familiar with the data and identify emergent themes. Next, the first author coded transcripts and fieldnotes to generate a preliminary list of inductive codes. We then applied the lens of the candidacy framework to the data set and coding scheme. Emergent codes were iteratively refined and integrated into a coding scheme using the dimensions of candidacy as an organizing framework (see Table 1). To support analytic rigor, both authors met throughout the analytic process to discuss emerging themes and to review the coding scheme. After finalizing the coding scheme, the first author coded the data set using Nvivo 11 software.

**Table 1.** *Dimensions of Candidacy and Corresponding Sub-Themes*.

Dimensions	Sub-themes	Representative quotes
Identification	• Finding relevant answers in an information sea	“[It’s] so overwhelming, all the stuff that you get.”
	• Perceptions of service suitability: Finding the right fit	“I can’t see him doing it.”
Navigation	• Continuous and complex navigation work	“You’re continually coming up with new ways to manage different things.”
	• The impacts of navigation work	“You have no idea how that crushes things.”
Appearances and Adjudications	• Age-restrictions and candidacy	“Most of the stuff is for people over 65.”
	• Not impaired enough or too impaired	“They don’t provide all day care if … his ADL’s aren’t bad enough.”
Operating Conditions	• A fragmented system	“I think it definitely is a problem that’s best dealt with by multidisciplinary teams.”
	• Representation and visibility	“Yeah, we’re silent…'Cause no one knows we exist.”

For the purposes of this paper, our analysis focused on the process of gaining access to services, and therefore does not include the dimension ‘*offers and resistance’,* which refers to the decision to accept or reject a service once access has been granted. Given the significance and complexity of this topic, and the volume of data we collected, we chose to conduct a separate analysis of participants’ decisions to accept or decline service offerings.

## Results

Participants accessed, or attempted to access, a variety of support services over time such as information support and referral; psycho-educational services (e.g. dementia education sessions); peer support groups; adult day services; day hospitals; home care services; recreational programs; volunteer programs; and mental health services. In the following sections, we present our analysis of access barriers and facilitators for each dimension of candidacy and corresponding subthemes (Table 1).

### Identification

In order to initiate access to support services, individuals or their carers must first identify themselves as a candidate for a particular service (Dixon-Woods et al., 2006). Our analysis shows that their ability to accomplish this work was contingent on their access to relevant information, as well as their perception of service suitability.

**Finding Relevant Answers in an Information Sea.** Overall, participants were knowledgeable about local services. Still, they found it difficult to access relevant information when they needed it. Many family members felt that information resources were geared towards older adults and not representative of the issues that affect people with young onset dementia or their families such as employment rights and financial benefits. Family members commonly described information they received as overwhelming, but also lacking. For example, one participant did not realize his wife was eligible for a disability benefit, even though he had received information about the benefit as part of a larger information package:

“*The thing that would have been really helpful to have sooner is the disability pension information. I actually did get it from Alzheimer Society, but I didn't realize I had received that information …[It’s] so overwhelming, all the stuff that you get…I do remember being encouraged to go to the different homes and start making a plan about where, you know which personal care home is right, but that's - that can be many years down the road, and it's in some ways too early for that. But the disability pension, that's right away*. (P08, Spouse)

One participant highlighted some of the additional barriers people with dementia face when accessing information resources. When asked what information he thinks is important for people with dementia to receive, this participant, along with his wife, responded:

Person with dementia (P05): *“When we had the diagnosis, I do not know. I did not understand it a lot so I do not know - did we get the right information? I think we got some information, did not we?”*

Spouse: *“Uh huh.”*

Person with dementia (P05): *“From that group?”*

Spouse: *“Right and from [the doctor], he recommended a book but you did not read it.”*

Person with dementia (P05): *“Reading for me, this is my reading: magazines, sports. I can tell you who’s playing hockey right now. It’s funny I can do that, but I can’t - reading a book is - it just does not.”*

Given the barriers to accessing and retaining information for some people with dementia, receiving written materials or attending time-limited educational sessions may not be adequate. Receiving information as part of an ongoing relationship with a knowledgeable provider or peer supports may help people with dementia overcome these barriers.

**Perceptions of Service Suitability: Finding the Right Fit.** Dixon-Woods et al. (2005) argue that the process of identification is influenced by perceptions of the quality and suitability of services. When considering the suitability of services, family members commented on the willingness of their relative to engage with services, the presumed characteristics of service users, and the extent to which a service or program was inclusive of people with dementia. When considering the suitability of a service, participants often compared their own (or their family members’) attributes to an imagined typical service user. Those who felt they differed from typical service users in terms of age, health condition, abilities, or interests, were less like to identify themselves as a potential candidate for that service. In the following quote, a family member described why she felt adult day services would not be a good fit for her husband:

*“I do not think he would [be willing to attend] and I do not even know if there are any day programs that would cater to somebody his age. You know, most of them cater to older seniors. And I know cause my mom had done that a few times, you know, and they come, they pick you up on a bus and take you to this day program. I can’t see him doing it.”* (P16, Spouse).

Family members and people with dementia also recounted attempts to find services in line with their interests. For example, participants had searched for volunteer opportunities, and programs that catered to their particular interests (e.g., woodworking, choir and exercise). In the quote below, a participant with dementia spoke of her desire to be part of a choir:

Person with dementia (P07): “*That would be nice, a choir*.”

Interviewer: “*That would be really nice, that’s a great idea. I like that idea.”*

Person with dementia (P07): “*I can sing all the songs from my past [laughs].”*

Spouse: *“And you are still able to learn new ones.”*

Interviewer: *“So music and singing, yeah.”*

Person with dementia (P07): *“My favourite.”*

However, participants had a difficult time identifying opportunities or recreational programs that were inclusive of people living with dementia. In the absence of a dementia-friendly choir, the participant above did not consider joining a local choir. Other family members researched recreational and volunteer opportunities that reflected their family member’s interests, but decided the program was unsuitable due to a lack of support and/or accommodations for people living with dementia.

## Navigation

Our analysis found that people with young onset dementia and their families were engaged in a continuous cycle of demanding navigation work. As needs changed, families struggled to find, access and coordinate services. The level and complexity of this work placed a significant burden on family members and, in some cases, prevented them from accessing needed supports.

**Continuous and Complex Navigation Work.** Family members engaged in a wide range of navigation work such as researching local resources, negotiating with gatekeepers, filling out applications, performing bureaucratic procedures, care planning, care coordination, and advocacy. Without specialized young onset dementia services, participants struggled to find and access supports. As one spouse commented: “*We did not really find any programs. We searched high and low and there was just nothing out there. There’s tons of stuff for over 65 but nothing for under 65*.” (P10, Spouse).

Family members and providers also highlighted the need for service coordination. Without access to ongoing care coordination, families struggled to manage change and piece together supports within a fragmented care environment:

*“It just keeps changing and so your grief just continues to change along with it and it never seems to end; and you are continually coming up with new ways to manage different things, from her leaving, to getting dressed, to her medication, to having someone there when I am at work, to every aspect of our life.”* (P03, Spouse).

Providers emphasized the need for navigation supports and access to a key health or service provider who can assist with care coordination over time:

*“I think there’s a need for more navigation and more support in primary care. Like, doctors unfortunately just do not have the time to -- you know, they can talk about dementia and they can do tests and screens, but that more psycho-social support? I think there’s a need for that.”* (P26, Provider).

**The Impacts of Navigation Work**. The amount and difficulty of navigation work posed a significant burden on families. Many family members were juggling caregiving, paid work and caring for their children. Some participants were dealing with mental health issues related to the impact of dementia and the stress of caregiving. The accumulative demands of navigating fragmented services added to an already difficult family situation. Family members frequently described their navigation efforts as time consuming, stressful, exhausting, overwhelming and frustrating. In the following quote, for example, a family member described her frustration after her attempt to arrange a volunteer opportunity for her husband fell through:

“*We went and had our meeting and then it turned out they had nothing for him to do and I'm like, oh my God do you know how much work it was for everybody involved to figure all this out and to get him… agreeable and excited about it? Like you have no idea how that crushes things because now if another day program comes up how am I ever gonna get him in there? It's frustrating*.” (P10, Spouse).

In some cases, family members avoided accessing services if the work required to access it was too onerous. As a result, those who are most in need of support may have a harder time accessing resources:

“*Part of the reason why I didn't make it to the support group is that like if you're younger and working and have a young family of your own and you're that so-called sandwich generation, like how do you attend a group with any regularity? How do you take advantage of those kinds of supports…But in some ways it's like all the steps you have to take just to get to the point where you're able to access it, or all the other things that have to take place in your own life in order to be able to take advantage of something. It's hard when you otherwise don't have time to do that*.” (P18, Adult son)

### Appearances and Adjudications

The constructs appearances and adjudications draw attention to the ways that candidacy is asserted, sustained or diminished through interactions between service users and providers (Dixon-Woods et al., 2006). In their attempts to access services, participants were confronted with age-restrictions and eligibility criteria that disqualified the person living with dementia for being either not impaired enough, on the one hand, or too cognitively and behaviorally complex, on the other.

**Age-Restrictions and Candidacy.** Participants encountered age-restrictions that prevented them from accessing a range of community supports including some adult day services, day hospitals, and mental health services. As one family member commented: “*We did have a case worker for a while but she mostly dealt with people over 65 and most of the stuff is for people over 65*.” (P20, Spouse). One family member discussed her attempt to get her husband into a day hospital program for people over 65 that was located at her workplace. Without other options to provide him company during the day, she decided to retire early:

*“It would've been wonderful if [my husband] could’ve gone to the day hospital … I could’ve continued working … Those supports were not there… The occupational health care nurse went to the geriatrician and said: ‘Can they make an exception?’. He would've done it, but the rules up above said no. So it’s frustrating*.” (P15, Spouse).

**Not Impaired Enough or Too Impaired.** In addition to age-restrictions, participants encountered restrictive eligibility criteria that categorized the person with dementia according to their cognitive and functional status, behavioural support needs, and family circumstances. Family members recounted being denied access to supports because their relative with dementia was either not “impaired enough”, or “too impaired”.

Accessing home care proved particularly challenging. Family members commonly explained that their relative's impairments did not meet a service's eligibility criteria geared towards functional limitations and safety considerations:

“*I ended up just retiring because I didn’t know what to do with him and when I talked to home care it was like well they'll come in and do pills or they'll come in and give him a bath but there really was nothing there for me to work and for him to stay home … I was told by home care that they only provide services, they don’t provide all day care if … his ADL's aren’t bad enough.”* (P15, Spouse).

Family members also described instances when their relative with dementia was disqualified from services for being “too cognitively impaired” or behaviourally complex. For instance, one family member discussed his mother’s disqualification from an adult day program:

“*She was eventually told that she couldn’t return because her attitude was too aggressive towards the staff which is really frustrating … so unless you’re of the demure, placid, laid-back type of person with dementia these programs aren’t accessible to you.*” (P18, Adult son).

Similarly, a service provider who coordinated support groups for people living with dementia described the challenge of accommodating clients as their condition progresses and behaviours change. Her comments highlight the limited and fragmented structure of community-based dementia services, which can result in the exclusion of people with dementia who are regarded as “inappropriate”:

*“There is kind of a gap … if somebody is not appropriate for a [support] group anymore because it’s for early-onset, so once they no longer think they have the disease, they wouldn’t qualify, if they need assistance in the bathroom, … if they start to become inappropriate or you know, they don’t have an hour and a half attention span anymore, things like that ... But what I sometimes come across, because I would have to be the one to tell people they were no longer appropriate, was if they didn’t want to go to an adult day program or in some cases they were no longer appropriate for an adult day program… and let’s say they weren’t going into a personal care home yet because the waiting list was too high, then family would say to me, ‘Well what is there for them?’”* (P35, Provider).

### Operating Conditions

This category refers to the organizational and broader structural factors that impact the local production of candidacy (Dixon-Woods et al., 2006). Our analysis found that service fragmentation and the invisible status of young onset dementia created structural barriers that constrained access to services and supports.

**A Fragmented System.** In the present study, support services were fragmented, time-limited, and delivered by a patchwork of health care providers, public sector services, non-profit organizations, and for-profit agencies. Without access to case management or coordinated services, participants struggled to manage change.

Providers also identified a need for coordinated, multidisciplinary services. The lack of integration constrained providers’ ability to support patients and families:

*“[Specialists are often set up as] solo practices where it is just the neurologist and a receptionist, and, you know, maybe you’d be lucky if you were in an outpatient department of a hospital, but you still might not have a nurse anywhere to be seen and you absolutely do not have a social worker, so if anyone is going to phone home care it has to be you, the consultant. So, I mean I think it definitely is a problem that’s best dealt with by multidisciplinary teams.”* (P25, Provider).

At the organizational level, access to services was also affected by resource limitations within the social care sector. Providers connected age-restrictions and narrow eligibility criteria to the scarcity of resources and resulting strain on services. One provider, for example, commented on the shift within a geriatric outreach service to discontinue accepting clients under 65 with dementia:

*“There was a time where … people could be seen even though they were under 65… But I think now it’s a pretty hard and fast rule that under 65, not being seen … I recognize that that comes from limited resources and they have to draw the line somewhere because they were feeling burdened by just too many people. The challenge is that I think the people who often times have the highest need are these families where someone has early-onset because there’s more complexity and there’s more need for support and resources*.” (P29, Provider).

**Representation and Visibility.** Underpinning access barriers was a lack of recognition and representation of young onset dementia at organizational and policy levels. Participants were forced to navigate systems and services that do not formally recognize their rights and needs. Age-restrictions and eligibility requirements geared towards older adults with functional limitations excluded people with young onset dementia from critical services, undermining the right to equitable care. Information resources and service offerings were also geared towards older adults and not representative of the conditions, characteristics, and support requirements of younger individuals. In the following quote, a service provider commented on the limited representation of young onset dementia within dementia care resources and programming:

“*What I hear from the families … the feeling is that they are not well represented in the dementia world. So whether it even be educational materials, seminars, workshops, things like that that are designed for families where there’s a dementia or even preventing dementia, that they don’t feel well represented by that. The pictures and you know all the examples are often of people who are much older and it’s still sort of thought of as, you know, a disorder of aging which it is in a lot of ways*.” (P29, Provider)

Some participants also pointed out that certain forms of dementia receive less attention than others. For example, a participant with Lewy Body dementia and his spouse commented on the predominance of Alzheimer’s disease and relatively invisible status of less common causes of dementia:

Spouse: “Not every person with dementia has a memory issue. They do not leave their keys in the fridge, you know? [My husband] has never put his keys in the fridge you know?”

Interviewer: “So do you feel like you’re not really represented?”

Spouse: “Oh, yeah. Huge, huge, huge.”

Person with dementia (P09): “Yeah, we’re silent.”

Spouse: “Oh, believe me I know.”

Person with dementia (P09): “‘Cause no one knows we exist.”

## Discussion

Despite growing recognition of the need to expand supports for people with young onset dementia, specialized services remain rare (Carter et al., 2018; Mayrhofer et al., 2018). Previous studies suggest that people with young onset dementia encounter barriers within mainstream dementia services (Cations et al., 2017; Greenwood & Smith, 2016); however, the social and contextual factors that influence service access have received limited attention. Using the lens of the candidacy framework, this study contributes to a more comprehensive understanding of multifaceted influences on service access from the perspectives of people living with young onset dementia, family members and service providers.

According to Dixon-Woods et al. (2006), the process of accessing services begins with identifying oneself as a candidate for a particular service. In line with previous studies (Cations et al., 2017; Ducharme et al., 2014; Millenaar et al., 2016), we found that problems finding relevant information constrained access. In addition, without clearly marked young onset dementia services, participants struggled to determine suitability. Even when services catered to an identified need such as respite or social support, participants did not identify themselves as candidates if they felt they differed from other users in terms of their age, life circumstances, abilities or interests. These findings affirm the recommendation by the non-profit organization, Young Dementia UK, for all dementia services to clearly communicate whether and how they accommodate people with young onset dementia or their families (Young Dementia Network Steering Group, 2020).

Accessing services requires work and resources on the part of service users (Dixon-Woods et al., 2006). A key finding of this study is that the level and complexity of navigation work required to access services created significant barriers and contributed to family stress. With limited support from providers, families struggled to identity needs, and to find and access services. Providers also felt constrained in their ability to assist with navigation due to resource limitations and a lack of care coordination. For family members, the time and effort required to navigate services compounded an already stressful family situation. In some cases, the level of work required to access services was prohibitive, causing participants to delay or avoid seeking services they considered valuable.

Age-restrictions have been previously identified as a key barrier to service use among people with young onset dementia (Cations et al., 2017). In addition to age-restrictions, people with dementia encountered gatekeeping and eligibility criteria that categorized them based on their age, cognitive and functional impairments, behaviour, support needs, and family circumstances. These adjudication processes often constructed people with young onset dementia as inappropriate users for being “not impaired enough” or “too impaired”. Participants’ candidacy appeared to be particularly constrained by restrictive eligibility criteria geared towards individuals with functional limitations, as well as the practice of excluding people categorized as behaviourally challenging. Given that behavioural changes are common among people with young onset dementia (O’Connor et al., 2021), these exclusionary practices may disproportionately affect people with young onset dementia and compound access barriers among those most in need. To promote equitable access, services should examine eligibility criteria to determine whether it disproportionately excludes younger people with dementia.

Our findings also show that organizational contexts constrained access in important ways. Fragmentation and resource scarcity across the dementia care sector diminished candidacy and compromised continuity of care. Despite growing calls for comprehensive, coordinated dementia supports (Carter et al., 2018; Mayrhofer et al., 2020), the services examined here were largely provisional supports delivered by a patchwork of public sector health and social services, non-profit organizations, and for-profit agencies. Without access to case management, families were tasked with identifying needs and accessing new forms of support. Mayrhofer et al. (2020) argue that though needed, specialized young onset dementia services are insufficient without broader service integration. Similarly, our findings underscore the need for coordinated service delivery to ensure continuity of care and to reduce the level of navigation work imposed on families.

Using the candidacy framework revealed access processes and vulnerabilities that had not been previously examined in the literature such as the level of work required to navigate services, restrictive eligibility criteria and fragmentation. In addition, our findings point to the need to extend the framework to account for the social and political status of user groups. The candidacy framework is underpinned by an interactionist orientation, which emphasizes the interactions between users, provider and institutions. Although this perspective recognizes the influence of social and political contexts on candidacy negotiations, the framework does not explicitly incorporate the social conditions that influence access (Chase et al., 2017). Our findings indicate that the status and visibility of people with young onset dementia influenced the construction of candidacy. With one exception, none of the services examined here formally recognized young onset dementia as a user group. This invisible status constrained candidacy, as participants were forced to navigate systems and services that do not formally recognize their rights or support requirements. For example, age-restrictions and eligibility requirements geared towards older adults systematically excluded people with young onset dementia, jeopardizing their right to equitable care. These exclusionary practices reflected and reinforced the invisible status of young onset dementia, illustrating the connection between candidacy and the status of user groups.

### Limitations

The results of this study should be considered in light of several limitations. First, the perspectives included in this study may not be transferable to other people living with young onset dementia and their families. Without dedicated services for people with young onset dementia, or a specialized dementia clinic in [city], recruitment was challenging. The study sample was relatively small, and all family members and participants with dementia were White and born in Canada. The lack of diversity is particularly notable given the diversity of [city’s] population. In addition, all participants with dementia had spouses who were actively involved in their care. As such, the study did not capture the experiences of people with different family structures or dynamics, such as those who are single. Recruiting participants from diverse backgrounds is a widespread challenge in dementia research (Dilworth-Anderson & Cohen, 2010). Future research that incorporates a range of strategies to promote diversity and inclusion is needed to capture the diverse experiences of people with young onset dementia across different family structures and categories of social location.

In addition, while all three participant groups offered insight into each dimension of candidacy, there were differences in terms of which dimensions were emphasized by each participant group. For example, family members provided the bulk of data concerning the process of identifying and navigating services, while interviews with providers elicited organizational and contextual features. Participants with dementia provided rich information about their experiences, but they tended to focus on their experiences using support services, and provided less information about the process of gaining access to services. Given the focus of this paper, most quotes came from family members and providers. Participants’ accounts of their experiences within support services will be presented in a separate analysis.

## Conclusion

Applying the lens of “candidacy” revealed multifaceted barriers to service use among people with young onset dementia and their families. Without service coordination, participants were engaged in a continuous cycle of navigation work and diminishing candidacy, which generated stress for families. Candidacy was further constrained by the lack of tailored information for younger people and families, and eligibility criteria geared towards older adults with functional limitations. At the organizational level, there was a tension between the progressive nature of dementia, and a system characterized by fragmented and time-limited supports. Taken together, these findings highlight the need for coordinated, inclusive services as well as broader representation of young onset dementia in research, policy and practice.
